# *N. meningitidis* and *TLR* Polymorphisms: A Fascinating Immunomodulatory Network

**DOI:** 10.3390/vaccines4020020

**Published:** 2016-05-27

**Authors:** Elena Gianchecchi, Alessandro Torelli, Giulia Piccini, Simona Piccirella, Emanuele Montomoli

**Affiliations:** 1 Strada del Petriccio e Belriguardo, 53100 Siena, Italy; torelli@vismederi.com (A.T.); piccini@vismederi.com (G.P.); piccirella@vismederi.com (S.P.); emanuele.montomoli@unisi.it (E.M.); 2 Department of Life Sciences, University of Siena, 53100 Siena, Italy; 3 Department of Molecular and Developmental Medicine, University of Siena, 53100 Siena, Italy

**Keywords:** *Neisseria meningitidis*, invasive meningococcal disease, *TLR* single nucleotide polymorphisms, risk factors, immunological responses, meningococcal vaccines

## Abstract

*N. meningitidis* infections represent a global health problem that can lead to the development of serious permanent sequelae. Although the use of antibiotics and prevention via vaccination have reduced the incidence of meningococcal disease, our understanding regarding *N. meningitidis* pathogenesis is still limited, especially of those mechanisms responsible for IMD and fulminant or deadly septic shock. These severe clinical presentations occur in a limited number of subjects, whereas about 10% of healthy individuals are estimated to carry the bacteria as a commensal. Since TLR activation is involved in the defense against *N. meningitidis*, several studies have highlighted the association between host *TLR* SNPs and a higher susceptibility and severity of *N. meningitidis* infections. Moreover, *TLR* SNPs induced variations in immunological responses and in their persistence upon vaccination against meningococcal disease. In the absence of mass vaccination programs, the early identification of risk factors for meningococcal disease would be recommended in order to start immunization strategies and antibiotic treatment in those subjects carrying the risk variants. In addition, it could allow us to identify individuals with a higher risk for severe disease and sequelae in order to develop a personalized healthcare of high-risk subjects based on their genomic profile. In this review, we have illustrated important preliminary correlations between *TLR* variants and meningococcal susceptibility/severity and with vaccine-induced immune responses.

## 1. *N. meningitidis* Infection and TLR Signaling Pathway Activation

*Neisseria meningitidis* (*N. meningitidis*) is a Gram-negative bacterium, which usually colonizes the human nasopharynx in about 10% of healthy individuals. Only a low percentage of subjects carrying *N. meningitidis* develops invasive meningococcal disease (IMD) with its highest incidence in children less than 2 years old. IMD shows different clinical presentations ranging from meningitis, fulminant shock or deadly septic shock [1]. Case fatality rate (CFR) varies from 4%–6% in cases of meningitis, reaching up to 40% in presence of septic shock [2], whereas serious long-term complications can be developed in up to 20% of IMD survivors. They include hearing impairment, motor deficits, amputations, hydrocephalus, mental retardation, neurologic and behavioral problems [3].

*N. meningitidis* infection induces the release of antibodies from the host’s immune system within 7–10 days. Most of the cases of meningococcal disease (MD) occur during the first week from the colonization of the nasopharynx, but they do not evolve into invasive meningitis, despite the absence of antibodies [4,5]. Antibodies represent an extremely efficient mechanism of defense against invasive meningococci. Consequently, subjects at higher risk of IMD onset are children in their early years of life, since this group shows a reduction in maternal antibodies and has not completely developed the defense mechanisms based on antibody production. Thus, a fundamental role in the protection towards *N. meningitidis* is played by innate immunity [6]. The mechanisms responsible for meningococcal sepsis, which is the result of complex interactions occurring between bacteria, coagulation, inflammatory and immunological responses of the host, are still largely unknown [7]. The clinical outcome of IMD is influenced by several factors, such as meningococcal virulence factors and host genetic [2,8,9,10,11,12,13]. The different susceptibility and disease outcome of individuals to MD could be due to genetic variants, including single nucleotide polymorphisms (SNPs) in host immune genes [9,13,14], which encodes for pathogen recognizing receptors (PRRs). These genetic variants might be responsible for an abnormal immune response characterized by a reduced or increased inflammation and thus they could alter recognition and clearance of bacteria [15]. This may explain why, although 10% of healthy individuals are estimated to carry the bacteria as a commensal in their upper respiratory tract, only a limited number of subjects experience meningococcal septic shock [16]. Furthermore, it has been hypothesized that innate immunity, which is phylogenetically older than the acquired immunity and represents the first defense against pathogens, could play a critical role in the protection against infections. A higher susceptibility and severity of infections caused by *N. meningitidis* are indeed related to alterations in the innate immune mechanisms rather than to mutations involving the acquired immune system [16]. Increasing evidence has showed that the presence of SNPs in *Toll-like receptor* (*TLR*) genes can represent a risk factor for IMD onset [2], as described also for other infectious diseases [17,18,19,20]. TLRs play a fundamental role in the recognition of pathogen associated molecular patterns (PAMPs), such as lipopolysaccharide (LPS), bacterial lipoproteins or unmethylated Cytosine-phosphate-Guanine (CpG) motifs, sensed by TLR4, TLR2 and TLR9, respectively [21,22,23]. Their activation promotes the downstream signaling pathway [23]. A correlation between *TLR* genetic variants and IMD susceptibility or complications has been hypothesized forasmuch. Although their activation is necessary for bacteria clearance, it can damage healthy cells causing adverse side effects [24]. Even though the immunological and inflammatory processes induced by the activation of TLR signaling pathway are under a strict negative regulation, their abnormal up-regulated activation could have deleterious effects on the host and be implicated in meningococcal sepsis. Furthermore, in certain conditions TLRs are implicated in the pathogenesis of autoimmune disorders [23].

## 2. *TLR* Genetic Variations in Host Response to Meningococcal Infection

TLRs recognize PAMPs derived from invasive microorganisms, such as bacteria, viruses, fungi and protozoa. After the ligation to PAMPs, TLRs induce the activation of the downstream signaling cascade by interacting and recruiting several adaptor molecules [23]. The activation of TLR pathway leads to the induction of type I interferons (INFs) and inflammatory cytokines, chemokines and co-stimulatory molecules [21]. Recently *TLR2*, *TLR4* and *TLR9* SNPs have been hypothesized to be associated with a predisposition to MD.

### 2.1. TLR4 SNPs

TLR4 plays an essential role in LPS recognition. Two SNPs affecting *TLR4*, Asp299Gly (D299G; rs4986790) and, to a lesser extent, Thr399Ile (T399I; rs4986791) are associated with Gram-negative sepsis and other infectious diseases. *TLR4* SNPs are found in humans with a frequency of about 6%–10% [25,26,27,28].

*TLR4* Asp299Gly SNP causes the substitution of an aspartic acid with a glycine at the amino acid 299 inducing the modification of the extracellular region of the receptor and the onset of functional defects [2]. The relevant role played by TLR4 in the defense against bacteria has been demonstrated both *in vitro* and *in vivo* studies, although several studies demonstrated species-specific differences in the recognition of lipid A portion of LPS by TLR4/MD-2. Experiments on C3H/HeJ (Lpsd) mice, characterized by a genetic *TLR4* altered expression, showed the influences of the host genome on meningococcal infection susceptibility upon injection with *N. meningitidis* serogroup C [25]. Particularly, Lpsd mice were characterized by a 1000-fold higher proliferation of bacteria with persistence of bacteremia for days before the infection was cleared, whereas the other mouse strains (Lpsn mice) experienced a shorter duration of bacteremia [25]. The study conducted by the group of Poltorak on C3H/HeJ and C57BL/10ScCr mice confirmed the critical role played by TLR4 in the protection against Gram-negative infections. In this study, a missense mutation within exon 3 caused a change in the cytoplasmic domain of TLR4, which was responsible for a defective LPS signaling. Consequently, *TLR4* mutations increased the susceptibility to Gram-negative infections and to the onset of sepsis [29]. In agreement with the results obtained in mice, the airway epithelial cells collected from subjects carrying *TLR4* SNPs (Asp299Gly and Thr399Ile) showed a diminished response to LPS and a diminished TLR4 expression [26]. Moreover, patients carrying *TLR4* variants were characterized by a significant higher incidence of Gram-negative infections [28,30]. The Asp299Gly polymorphism has been associated to several infections and autoimmune disorders [28], whilst the presence of Thr399Ile polymorphism was shown to induce a weaker hyporesponsiveness to LPS or even no alteration [31]. Recently, it has been demonstrated that Asp299Gly and Thr399Ile *TLR4* SNPs were not able to influence TLR4 expression, but the presence of Asp299Gly induced a defective recruitment of adaptor molecules involved in the signaling pathway and in pro-inflammatory cytokine’s synthesis [28].

However, contrasting results regarding the possible association between Asp299Gly SNP and IMD susceptibility have been reported [32,33,34]. Particularly, Thr399Ile SNP was not evaluated in the study conducted by Allen since this variant was not present in the population analyzed [33], whereas no association was found between group A MD and Asp299Gly. However, it is possible that no correlation was observed since this investigation was conducted on Gambian children and for this reason, it cannot be compared to the other studies performed on European populations [16]. Although the frequent *TLR4* (*TLR4B*) SNP and variant *TLR2* alleles were not associated with susceptibility to IMD, a higher frequency of rare and missense TLR4 variants was observed in MD patients by the group of Smirnova [34].

Since a marked and prolonged bacteremia upon experimentally induced infection has been demonstrated in mice carrying a defective TLR4 expression [25], Read and colleagues [32] have investigated whether the presence of *TLR4* Asp299Gly SNP in humans could enhance their susceptibility to MD or influence its clinical manifestations. 1047 MD Caucasian patients and 879 healthy controls were genotyped. Although patients showed a slight increase in SNP frequency compared to that observed in controls, no significant correlation was observed between Asp299Gly SNP and susceptibility to, or severity of, the pathology. It has been supposed that the absence of an association in such fundamental TLR, involved in LPS sensing, and whose variants are responsible for endotoxin hyporesponsiveness in humans [26], could be due to the replacement of TLR4 function by other TLRs or components belonging to TLR signaling pathway [32]. Conversely, the two *TLR4* variants (Asp299Gly and Thr399Ile) were demonstrated to constitute risk factors for IMD in infants less than 12 months of age, since they presented an increased frequency of these allele variants [35]. A following study conducted by the same group [6] also found a significant association between IMD subjects heterozygous for Asp299Gly variant and lethal outcomes. This correlation was even stronger in children aged less than 2 years, wherein mortality was 4-times higher. The need of ventilation support was also enhanced in this age group [6]. The retrospective case-control study conducted by the group of Biebl [36] on Caucasian patients that survived MD confirmed the results previously obtained by Read [32]. Moreover, they investigated the putative correlation between IMD and the cluster of differentiation (CD)14 C-159T SNP for the first time. The latter is a PRR, fundamental in innate immunity allowing the transfer of bacterial ligands, such as LPS, to the signaling complex made up of TLR4 and myeloid differentiation factor 2 (MD-2). However, no association was observed [32]. TLR genetic variants were not only able to influence the clinical presentation and outcome of MD, but also to damage neurons and contribute to the inflammatory process of the cochlea as demonstrated by the positive correlation between post-meningitis complications such as hearing loss and TLR4 +896 SNP. The risk of deafness was even higher in co-presence of TLR2 +2477 wild type (WT) or of TLR9 −1237 SNP [15]. In a following study conducted by the same group [37], not only an association between TLR4 +896 (D299G) SNP and meningococcal meningitis (MM) onset was reported, but also a strong correlation with the combined carriage of this TLR4 SNP and TLR2 +2477 (rs5743708) was observed in a large Dutch Caucasian cohort. A genetic trait involving the combined carriage of TLR4 and TLR2 SNPs has been found in other pathologies, such as lupus, malaria and tuberculosis, but this study was the first to find a correlation with MM susceptibility [37]. The contrasting results regarding the putative association between TLR4 +896 and MD previously published could be due to the different ethnic populations analyzed or also to different serogroups responsible for MD that may induce a peculiar PRR stimulation. In fact, whereas no correlation was reported from the analysis conducted on a cohort of Gambian children affected by MD belonging to serogroup A [33], van Well analyzed a cohort in large part constituted by serogroup B cases and with just one case caused by serogroup A [37]. The group of Tellerìa-Orriols [14] also genotyped *TLR4* Asp299Gly SNP in 59 IMD Caucasian children and 66 healthy subjects by using restriction analysis observing that *TLR4* SNP frequency was more than 2-times higher frequent in IMD patients compared to controls [14]. In addition they reported that 50.8% of IMD patients carried at least one copy of *TLR2* (R753Q, rs5743708) and *CD14* (c.−159C > T, rs2569190) risk alleles, the latter described as a risk factor for the first time [14].

### 2.2. TLR9 SNPs

Another TLR, which has a critical function in the defense against meningococcal sepsis as demonstrated in knockout (KO)-mice, is represented by TLR9 [38]. As previously described, TLR9 SNPs have been correlated with the clinical outcome and complications in patients affected by MD maybe through an altered recognition of CpG motifs [15]. Whereas TLR9 +2848 SNP conferred protection against MM, probably through the up-regulation of immune response, the carriage of TLR9 haplotype I was strongly associated with an elevated MM susceptibility in children [39]. These results were also confirmed later by a study performed by the same group. In addition the presence of TLR9 (−1237 and +2848) SNPs correlated with prevention against bacteremia and with significantly elevated cerebrospinal fluid (CSF) leukocyte levels during MM compared to WT carriers. However, no association with clinical sepsis was observed. The reduction of bacteremia could be due to a stronger immune response in the CSF of the host. It has been demonstrated that the stimulation of cells carrying TLR9 −1237 C allele promoted the transcription of the gene by increasing the affinity of nuclear factor κB (NFkB) to TLR9 promotor gene and thus to higher levels of chemokines and cytokines [40]. Since no effect was observed in cells under basal conditions, it has been hypothesized that this genetic variant may be correlated with severity, whereas it could exert no effect on MM susceptibility [39].

### 2.3. TLR2 SNPs

Also *TLR2* (p.R753Q, rs5743708) SNP, located in the Toll/IL-1 receptor (TIR) region of *TLR2* and playing an essential role in TLR signaling pathway as well as in the formation of dimers with other TLRs [41,42], was significantly associated with IMD compared to controls. In fact, it was found that at least one copy of this allele was carried by 59% of IMD patients [14]. The presence of this SNP induces a defective TLR2 dimerization and thus, it could be responsible for a reduced response against meningococcal porin B (PorB) mediated by TLR1 [43].

### 2.4. Defective TLR Signaling Pathway

In addition to the previous findings, even the presence of a defect in one molecule involved in TLR signaling pathway plays a critical role in the protection against Gram-negative bacterial infections [16,44]. Particularly, a patient who was affected by recurrent bacterial infections since her infancy presented two different mutations in IRAK-4 [44], which belongs to the IL-1 receptor-associated kinase (IRAK) molecule. Among the members belonging to IRAK family, IRAK-4 has the most critical function in the MyD88-dependent signaling pathway activated by TLR4, TLR2/1 and TLR2/6 [23]. The altered form of IRAK-4 was not able to recruit and activate the downstream signaling molecules. The subject was in fact hypo-responsive to LPS *in vivo*. Furthermore, she showed a defective abnormal inflammatory response as demonstrated by leukocyte hyporesponsiveness to LPS and IL-1 *in vitro* [44]. The group of Picard [45] found homozygous recessive IRAK-4 deficiencies in three unrelated patients presenting recurrent pyogenic infections. Interestingly, the substitution C877T of the *IRAK-4* cDNA was a common variant among two of the three patients analyzed by Picard [45] and the subject described by Medvedev [44]. These findings support the hypothesis that this site could constitute a hypermutable site or “hot spot” in this gene that may be present in other subjects affected by frequent bacterial infections [44].

## 3. The Influence of *TLR* SNPs on Vaccine-Induced Immunity

It is well known that vaccination responses are influenced by both environmental and endogenous factors, such as sex, age and genetic variations [46]. Studies on twins revealed the importance of genetic factors [47,48,49,50,51], contributing to 36%–38% of heritability, a percentage value evaluated as genetic variant ratio compared to total variance [52]. The ability to predict the responses to immunization on the basis of host genetic could constitute a critical factor to develop personalized vaccine strategies. [53,54]. Different types of meningococcal vaccines are currently available against the different serogroups (A, C, W, Y and recently also B) and they have been introduced in the routine immunization programs depending on the most common serogroups present in a specific country [55]. These formulations include polysaccharide-protein conjugated and plain polysaccharide vaccines. In the case of *N. meningitidis* serogroup B, meningococcal outer membrane vesicle (OMV)-based vaccines and recombinant protein vaccines are available [56].

As reported for mumps, rubella, measles, *bacillus* Calmette-Guérin (BCG), influenza A and hepatitis B vaccinations, marked inter-individual differences in the level and persistence of immunity upon MenC vaccine have been found, although the mechanism is not completely clear [57]. Recent studies have demonstrated that *TLR* allelic variants were able to induce variations in immunological responses following immunization against MD, as it was reported that their presence correlated with the antibody response [58,59]. Spector and colleagues [58] reported an altered antibody response to quadrivalent meningococcal conjugate vaccine (MCV4) in HIV-infected children carrying *TLR2* (-G2408A;rs5743708), *TLR4* (-A12874G;rs4986790) and *TLR9* (-T1237C;rs5743836) variants. The first two variants were able to enhance the first response to the vaccine against the four invasive *N. meningitidis* serogroups. This immunological response was evaluated as the achievement of a ≥4-fold increase in serum bactericidal antibody (SBA) response after 4 weeks from vaccine administration; the latter has a role on the long-term response (evaluated at weeks 28 and 72). No data were available on the influence of these SNPs in healthy children [58]. *TLR3* SNPs were able to influence the persistence of anti-measles [60] as well as anti-MenC selective antibodies both in infants/children and teenagers [61]. A following recent study conducted by O’Connor revealed that an exonic variant in *TLR3* correlated with MenC IgG levels in infants [59]. Since the activation of TLR signaling pathway is critically involved in the promotion of adaptive immune responses, several adjuvants added to vaccine formulations in order to enhance the immune reaction towards the immunogen, mimic TLR ligands. Although vaccine adjuvants have been supposed to be implied in triggering or increasing the development of the autoimmune/inflammatory syndrome [54,62] through the activation of several endosomal or surface TLRs, no convincing data support the presence of a correlation between vaccine adjuvants and autoimmunity [63].

## 4. Conclusions

Increasing data revealed the association between MD susceptibility and the presence of genetic variants in several host immune genes, such as those involving properdin, which regulates the alternative complement pathway, the complement, the mannose-binding lectin (MBL) and TLR pathways [14,24]. The present review is focused on SNPs affecting TLR signaling pathway, since its activation represents one of the primary mechanisms of defense against *N. meningitidis*. As demonstrated by several investigations, in addition to *TLR* SNPs, also mutations affecting the signaling components involved in the downstream transduction of this pathway exert an important role causing a deficient inflammatory response [44]. However, one limit of the published studies regarding *TLR* SNPs and their influence on MD is represented by the fact that they are in a restricted number, involving often limited cohorts of patients and controls. This fact might explain the reason why contrasting results were obtained in some cases. In this context, the advent of genome wide studies (GWS) conducted on meningococcal isolates might allow us to increase our knowledge of the complex interactions between TLR genotypes and the susceptibility or severity of MD, as recently demonstrated in two studies, which confirmed the critical role played by complement factor H (CFH) region polymorphisms in this serious infection [64]. A further breakthrough is represented by the advent of high-throughput sequencing technology, which has recently allowed us to obtain a detailed characterization of the B cell receptor (BCR) repertoire upon vaccination, unfeasible when the more conventional techniques of immunogenicity are used. This method has been applied in order to assess the response in adults immunized with a conjugate Hib/MenC/tetanus vaccine [65] and meningococcal ACWY plain polysaccharide or polysaccharide-protein conjugate vaccinations [66]. It is undeniable that vaccine immunogenicity is influenced by a variety of factors and among these, host genetic components play a non-secondary role [51,57]. Obviously, the advent of these new technologies constitutes an incredible enhancement that allow us to quantify immune responses to vaccinations which until now have been rather limited, measuring the levels of antibodies and, for several vaccinations, of cytokines [57].

Following a wider approach, the early identification of multiple biomarkers constituting risk factors for MD would be recommended in order to start vaccination strategies and antibiotic treatment in those subjects carrying the risk variants [13]. In addition, it could allow us to identify children with a higher risk for severe disease and sequelae, such as hearing loss [17] and, in this case, to develop a personalized healthcare of high-risk individuals through a selective pharmacological treatment based on their genomic profile [20,39,67,68]. Another critical point that should be investigated is represented by the hereditability of immune responses to a certain number of vaccines, including those for *N. meningitidis*. In this review, we have illustrated important preliminary correlations between *TLR* variants and meningococcal susceptibility or severity and also with vaccine-induced immune responses.

Further data are necessary to clarify and confirm these preliminary findings regarding the association between MD and *TLR* SNPs and genetically induced variations in vaccine responses in order to identify individuals at a high MD risk.
